# Incidence and Perioperative Risk Factors for Postoperative Delirium After Major Urological Surgery

**DOI:** 10.3390/diagnostics15243165

**Published:** 2025-12-11

**Authors:** Vesna Jovanovic, Sandra Sipetic Grujicic, Natasa Petrovic, Branka Terzic, Milos Lazic, Kristina Burgic Vidanovic, Nikola N. Ladjevic, Ivana Markovic, Nebojsa Ladjevic

**Affiliations:** 1 Center for Anesthesiology and Resuscitation, University Clinical Center of Serbia, Pasterova 2 St., 11000 Belgrade, Serbia; dencic.natasha@gmail.com (N.P.); terzic.branka@gmail.com (B.T.); m.lazic1003@gmail.com (M.L.); burgictina@yahoo.com (K.B.V.); ivamark84@yahoo.com (I.M.); nladjevic@yahoo.com (N.L.); 2 Faculty of Medicine, University of Belgrade, Dr Subotica 8 St., 11000 Belgrade, Serbia; sandra.grujicic2014@gmail.com; 3 Institute of Epidemiology, Faculty of Medicine, University of Belgrade, Dr Subotica 8 St., 11000 Belgrade, Serbia; 4 Clinic for Urology, University Clinical Center of Serbia, Resavska 51 St., 11000 Belgrade, Serbia; nikola.ladjevic@yahoo.com

**Keywords:** postoperative delirium, elderly patients, urological surgery

## Abstract

**Background**: Postoperative delirium (POD) is one of the most common surgical complications in elderly patients. This study investigated the incidence of and perioperative risk factors for POD following urological surgery. **Methods**: A total of 162 male patients aged ≥50 years undergoing elective major urological surgery under general anesthesia from May 2024 to March 2025 were included in this prospective observational study. Delirium was assessed using CAM-ICU twice a day for five postoperative days. Groups with and without delirium were compared, and perioperative predictors of delirium were analyzed. Multivariate regression analysis was used to identify independent risk factors for POD. **Results**: Overall, 16% of patients developed delirium during the follow-up period. Patients with POD were significantly older (mean age, 73.3 ± 5.2 years vs. 66.3 ± 7.2, *p* < 0.001), had more comorbidities, and lived in rural areas. Atrial fibrillation and COPD were particularly significant. The incidence of POD was higher in patients with mild/moderate alcohol consumption than in those who never drink. Analysis of intraoperative factors revealed a significant difference between groups in the presence of intraoperative hypotension and blood transfusion. Patients with delirium had more severe postoperative pain. Optimal cutoff values of age (≥67.5), number of comorbidities (≥2), preoperative MMSE score (≤25.5), and postoperative NRS score (≥4.85) were determined using ROC curves. The multivariate analysis identified age ≥ 67.5 years, COPD, mild/moderate alcohol consumption, preoperative MMSE score ≤ 25.5, intraoperative hypotension, and postoperative NRS score ≥ 4.85 as independent risk factors in this cohort. **Conclusions**: Considering that some of the above risk factors can be modified, it is necessary to emphasize that the prevention of POD is possible and should be one of the treatment priorities in older patients.

## 1. Introduction

Postoperative delirium (POD) is one of the most common surgical complications among elderly patients, and the incidence varies significantly depending on the surgical procedure [1,2,3]. The main characteristics of this syndrome are acute and fluctuating changes in consciousness, attention, perception, thinking, emotion, memory, psychomotor behavior, and sleep/wake schedule [4]. It often occurs within the first three postoperative days, with symptoms lasting from 1 to 3 days, and the clinical presentation is variable [5,6]. The patient may have different motoric subtypes of POD: a reduced level of activity or agitated, hyperactive behavior, and often the hypoactive form may go unrecognized if routine delirium monitoring is not used [7]. POD is associated with negative treatment outcomes, including increased length of hospital stay, treatment costs, and mortality [8,9,10]. Moreover, in many patients over the age of 60, cognitive deficits can persist for up to three months after surgery [11]. This syndrome is recognized by the American Geriatrics Society as a prevalent postoperative complication [8] that is strongly associated with different modifiable risk factors [12], and by influencing these factors during hospitalization, some cases can be prevented [13].

Few studies have investigated POD development after urological surgery [14], and the reported incidence among the urologic population varies among studies (1.7–47%) [15]. About 60% of all urological procedures are performed on elderly patients [16], making POD research very important in this surgical population. Identification of its specific predisposing factors can contribute to developing preventive strategies [17].

## 2. Materials and Methods

### 2.1. Study Population

Following approval by the Ethics Committees of the Faculty of Medicine, University of Belgrade, Serbia (22.05.2024. No. 25/V-2), and by the University Clinical Center of Belgrade, Serbia (26.10.2023. No. 576/11), we conducted a single-center, prospective, observational cohort study.

This study included men aged ≥ 50 undergoing elective open urological surgery at the Clinic for Urology, University Clinical Center of Belgrade, between May 2024 and March 2025. Written informed consent was obtained from all of the study participants. Patients were excluded from participation if they refused to participate, had pre-existing delirium or delirium on admission, had a history of alcohol and drug abuse, and had conditions that could have potential confounding effects, such as neurodegenerative diseases (Parkinson’s disease, Alzheimer’s disease), cerebrovascular disease (history of stroke or transient ischemic attack), or psychiatric disorders (depression, schizophrenia, bipolar disorder). We excluded patients with a diagnosis of dementia or severe cognitive impairment (MMSE score 0–17), severe vision impairment, or hearing disorder.

**Figure 1:**
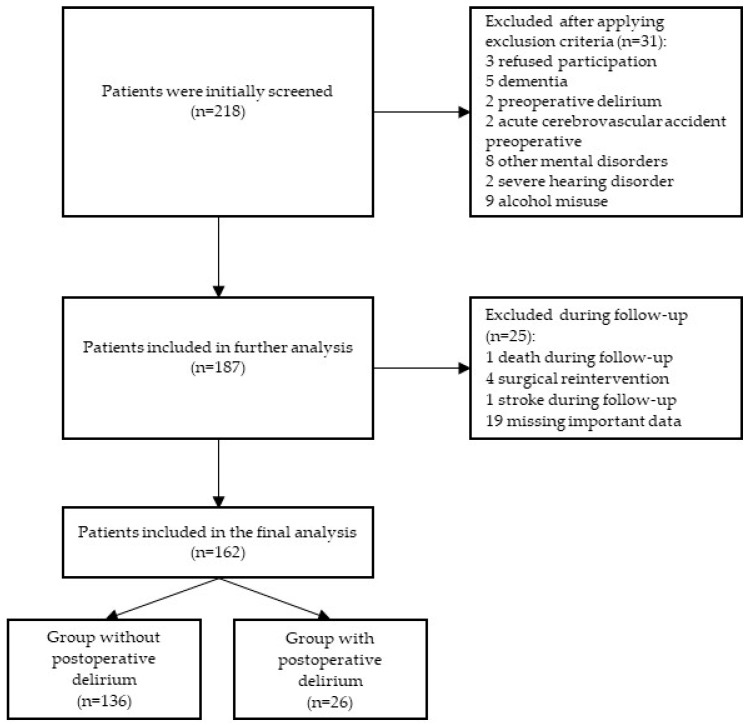
Study flowchart.

Figure 1 shows that, from a total group of 218 patients undergoing elective major urological surgery, 162 patients were included in the final study population.

### 2.2. Preoperative Assessment and Data Collection

Data of interest were collected in personal interviews and by reviewing medical records. Preoperative data included age, height, weight, body mass index (BMI), place of residence, marital status, education level, alcohol consumption, smoking, comorbidities and therapy. Alcohol consumption was determined based on the number of standard drinks per day. The severity of alcohol use is defined as mild to moderate drinking (2 drinks or less per day), binge drinking (5 or more standard drinks in 2 h), heavy alcohol use (5 or more standard drinks on any given day and more than 15 drinks in a week) [18,19]. Only patients who never drink alcohol and patients whose alcohol consumption is mild/moderate were included in this study. The cognitive status was determined using the Mini-Mental State Examination (MMSE) one day before surgery. MMSE is primarily a screening test for dementia and has been reported to be associated with postoperative delirium [20]. Scores range from 0 to 30, with 24 being a commonly used cutoff. However, a specific optimal MMSE cutoff score that would indicate possible occurrence of POD in patients of different ages has not yet been determined. Comorbidities are expressed as the absolute number of comorbidities [21,22].

### 2.3. Surgical Procedures and Anesthesia

All patients underwent one of the open urological procedures, namely, radical cystectomy, radical prostatectomy, nephrectomy or nephroureterectomy, under general anesthesia. Induction agents included propofol or etomidate and fentanyl, and muscle relaxation was achieved with rocuronium or cisatracurium. Anesthesia was maintained with sevoflurane in oxygen and air. Intraoperative analgesia was achieved with bolus doses of fentanyl and maintenance of muscle relaxation with bolus doses of rocuronium or cisatracurium. The surgery duration, intraoperative blood loss, blood transfusion administration, and amount of transfusion, as well as episodes of intraoperative hypotension, were recorded. Intraoperative hypotension was defined using the absolute values outlined in the Perioperative Quality Initiative (POQI) workgroup consensus statement [23]. At least one episode of hypotension (SBP < 100 mmHg or MAP <65 mmHg) lasting at least 5 min was recorded and analyzed [24]. Fluid management and titration of anesthetic dose were at the discretion of the attending anesthesiologist. Records were maintained regarding whether the anesthesiologist adjusted the dose of anesthetics and opioids using EEG monitoring or whether the drug titration was performed using routine parameters (the value of the minimum alveolar concentration, blood pressure, pulse, pupil width, presence of sweating, etc.).

### 2.4. Postoperative Pain Assessment and Management

Postoperative pain intensity assessment was performed using the numerical rating scale (NRS), a pain assessment tool where patients rate their pain intensity on a scale of 0 to 10, with 0 representing “no pain”, 10 representing the “worst pain imaginable”, and the pain intensity categories being mild (1–4), moderate (5–6), or severe (7–10) [25]. According to standard department analgesia protocol, all patients postoperative received a combination of metamizole and tramadol, or acetaminophen and tramadol. As a rescue analgesic, morphine 1 mg IV was used every 5–10 min until the pain intensity on the NRS decreased to ≤3. Pain intensity at rest was assessed 1, 2, 3, 4, 6, 8, 12, 16, 20, and 24 h postoperatively.

### 2.5. Diagnosis of Postoperative Delirium

Delirium was assessed using the Confusion Assessment Method for the Intensive Care Unit (CAM-ICU), a widely used diagnostic tool with high sensitivity and specificity in identifying delirium, during five postoperative days [26,27]. Assessments were performed by anesthesiologists twice daily between 7:00 and 10:00 am and between 07:00 and 22:00 pm. CAM-ICU assesses four key features: acute onset or fluctuating course, inattention, disorganized or incoherent thinking, and alteration in alertness. If criteria one and two are present and criterion three or four is present, the diagnosis of delirium is made. Before performing the CAM-ICU test, the level of consciousness or the level of sedation was assessed using The Richmond Agitation-Sedation Scale (RASS). Patients with a RASS score of −4 or −5 are not eligible for CAM-ICU evaluation. The diagnosis of delirium was made when the RASS score ≥ −3 and the CAM-ICU test was positive. If the RASS score was persistent between +1 and +4 during all assessments and the CAM-ICU test was positive, a diagnosis of hyperactive delirium was made; if the RASS score was persistent between 0 and −3 with a positive CAM-ICU test, hypoactive delirium was diagnosed. The mixed subtype is diagnosed when a patient has both RASS values [26,27,28]. For each patient diagnosed with delirium, a psychiatrist was consulted to confirm the diagnosis and to propose a treatment plan.

### 2.6. Statistical Analysis

For normal distribution data testing, the Kolmogorov–Smirnov and Shapiro–Wilk tests were used. To describe the variables of importance and depending on their nature, measures of descriptive statistics were used: frequencies, percentages, mean value (average), median, standard deviation (SD), and range. The statistical significance level was set at *p* < 0.05. In the case of multiple testing on the same data set, Bonferroni’s α-value correction was used (α_1_ = 0.05/3 = 0.0167). To test the differences between different outcomes (delirious and non-delirious) and depending on the nature of the examined parameters, the Pearson χ^2^ test, Fisher exact test, and Wilcoxon rank-sum test were used. To investigate the discriminative potential of parameters important for POD, the Receiver Operating Characteristics (ROC) curve methods and the Area Under the Curve (AUC; AUC ROC—Area Under the ROC Curve) according to the DeLong method were used. Logistic regression and likelihood ratio tests were used to examine the significance of the AUC ROC. Based on ROC analyses, the best cutoff values were set as values with maximum sensitivity and specificity. Moreover, for evaluating potential predictors of POD, univariate and multivariate logistic regression was used. The Odds Ratio (OR) with a corresponding 95% Confidence Interval (CI) was used for the description, while the likelihood ratio test and the Wald test were used to test the statistical significance of the factors in the regression model. The statistical analyses were performed with the program R (version 4.3.1 (2023-06-16 ucrt) “Beagle Scouts”; Copyright (C) 2023 The R Foundation for Statistical Computing; Platform: x86_64-w64-mingw32/x64 (64-bit)) (available at www.r-project.org; downloaded on 21 August 2023).

## 3. Results

### 3.1. Baseline Characteristics

A group of a total of 218 male patients age ≥ 50 years who underwent an elective open urological procedure was initially screened. After applying exclusion criteria, 31 patients were excluded: 3 refused to participate, 5 had dementia, 2 had preoperative delirium, 2 had acute cerebrovascular insult preoperatively, 8 had mental disorders (1 schizophrenia, 6 depression, 1 bipolar disorder), 2 had severe hearing disorder, and 9 had alcohol abuse. In the further course of this study, 25 patients were excluded from the final analysis: 1 patient died before the end of this study, 4 had surgical reintervention, 1 had a stroke during the follow-up period, and 19 were excluded due to missing important data. Finally, 162 patients were included in this study (Figure 1).

In this cohort study, among the 162 male patients, the majority were older than 67 years, lived in an urban area, were married, had a high school education, were non-smokers, and did not consume alcohol (Table 1). Patients generally had up to two comorbidities. The most common were the presence of hypertension (124/162 pts, i.e., 76.54%), chronic kidney disease (45/162 pts, i.e., 27.78%), and diabetes mellitus (32/162 pts, i.e., 19.75%; Table 1).

The mean preoperative MMSE score of the whole cohort was 27.9 (±2.17). This cohort included patients with the following procedures: 45 (27.78%) radical cystectomy, 51 (31.48%) nephrectomy, 11 (6.79%) nephroureterectomy, and 55 (33.95%) radical prostatectomy. The median surgery duration was 170 min (range 60–480 min), with a median estimated blood loss of 300 mL (range 50–1400 mL). Intraoperative EEG monitoring for anesthesia depth titration was used in 72 (44.44%) patients. An episode of intraoperative hypotension was recorded in 68 (41.98%) patients, and intraoperative blood transfusion was administered in 39 (24.07%) patients (Table 2).

### 3.2. Postoperative Delirium (POD)

The aim of this research was to identify factors significantly associated with the occurrence of POD, which was recorded in 26/162 (16.05%) patients during the follow-up period. The results of the comparison of all parameters between these two groups of patients (without POD vs. with POD) are shown in Table 1 and Table 2.

Patients with delirium were significantly older (*p* < 0.01; Table 1) and lived in rural areas (*p* < 0.01; Table 1). The incidence of POD was significantly higher in patients whose alcohol consumption was mild/moderate than in those who never drink (*p* > 0.01; Table 1). Regarding the presence of comorbidities, patients with POD had a significantly higher number of comorbidities (*p* < 0.01; Table 1), with a particularly significant presence of atrial fibrillation (*p* < 0.01; Table 1) and COPD (*p* < 0.01; Table 1). Moreover, patients with POD had a lower preoperative MMSE score (*p* < 0.01; Table 2).

According to the type of surgical procedure, the occurrence of POD was high after radical cystectomy (46.15%), followed by radical nephrectomy (38.46%), but less frequent after radical prostatectomy (11.54%) and nephroureterectomy (3.85%) (test for all types of operations: *p* < 0.05; Table 2). In all cases, delirium occurred within 3 days after surgery, lasting from 2 to 4 days. Of the 26 patients, 4 had hypoactive delirium, 10 had hyperactive delirium, and 12 had a mixed type.

An analysis of intraoperative risk factors revealed a significant difference between groups in the presence of episodes of intraoperative hypotension (*p* < 0.01; Table 2) and blood transfusion application (*p* < 0.05; Table 2). Patients with POD experienced significantly higher pain intensity during the first 24 h after surgery (*p* < 0.01; Table 2).

### 3.3. ROC Curve Analysis

The Receiver Operating Characteristic (ROC) curve methods were used to examine the discriminatory potential of numerical variables that showed a statistically significant difference between groups (Table S1 and Figure S1).

By categorizing numerical values according to ROC cutoff values, we defined new categorical variables and their association with the presence of POD, as shown in Table 3. The occurrence of POD was high in patients ≥ 67.5 years old (*p* < 0.01; Table 3), with two or more comorbidities (*p* < 0.01; Table 3), preoperative MMSE score ≤ 25.5 (*p* < 0.01, Table 3), and with higher postoperative pain intensity (*p* < 0.01, Table 3).

### 3.4. Univariate and Multivariate Logistic Analysis

Univariate and multivariate logistic regressions were performed to identify the potential risk factors of postoperative delirium (Table 4). First, this analysis was performed with all the significant factors that were assessed as significantly different in the analysis comparison between the delirium and non-delirium groups. The multivariate analysis identified age ≥ 67.5 years, COPD, mild/moderate alcohol consumption, preoperative MMSE score ≤ 25.5, intraoperative hypotension, and postoperative NRS score ≥ 4.85 as independent risk factors for postoperative delirium in this cohort (*p* = 0.036, 0.007, 0.001, 0.008, 0.002, and 0.001, respectively).

## 4. Discussion

In the present study, the incidence of POD was 16% (26/162 patients). The number of patients with delirium was relatively small, so the sample size may have been too small to identify all significant associations related to POD. The occurrence was highest after radical cystectomy (12/26 patients), followed by radical nephrectomy (10/26 patients), radical prostatectomy (3/26 patients), and nephroureterectomy (1/26 patients). The timing in our study was similar to that in previous studies, in which delirium most often occurred early in the postoperative course [21,22,29].

It is well known that advanced age is a risk factor for POD [30,31]. Although the mechanism of action is not known, it is believed that presence of comorbidities and reduced functional and cognitive capacity of elderly patients lead to an increased incidence of postoperative complications. Consistent with previous reports, our results demonstrated that older age was highly predictive of delirium. ROC curve analysis revealed age ≥ 67.5 years to be the optimal cutoff age for developing delirium, with a sensitivity of 88.5%, and the results show that the risk of POD is much higher in patients aged ≥67.5. The results from a meta-analysis by Gracie et al. showed that POD is another significant complication in the elderly, with an incidence of 7–56% among patients over 65 years [30]. In a meta-analysis that included a total of 8382 patients undergoing noncardiac surgery, researchers showed that patients aged 65–85 years were at high risk of developing POD (OR 2.67; 95% CI 2.16–3.29), and the risk in patients older than 85 years was 6.2 times higher than in those younger than 65 [31]. Although age is not a modifiable risk factor, the use of POD prevention strategies in older patients may influence both the occurrence and alleviation of symptoms in this population.

Data on the influence of certain variables such as the level of education, marital status, BMI, and smoking on the occurrence of POD are conflicting. These variables in our research did not prove to be significant predictors for POD, which is in accordance with the results of numerous studies [21,22,29]. However, some studies have identified these variables as risk factors for POD [31,32,33]. The occurrence of POD in patients who abuse alcohol is well known. Regarding drinking, this study included patients who never drink and who occasionally (mild/moderate) drink alcohol. The results showed that even mild/moderate alcohol consumption in elderly patients significantly increases the risk of developing POD (OR = 20.73 95% CI 3.67 to 117.03 *p* < 0.001). In contrast to these results, Togoni et al., Xue et al., and Large et al. did not show a significant relationship between active alcohol consumption and the occurrence of POD after urological surgery in the elderly [21,22,29].

Identification of specific comorbidities which are associated with POD can help in its prevention through adequate preoperative optimization of these conditions. In this study, patients who had two or more comorbidities had a higher incidence of POD. Two comorbidities were significant: atrial fibrillation and COPD. Further analysis found that COPD was an independent risk factor for POD. It can increase the occurrence of POD by increasing systemic inflammation and postoperative respiratory complications [34]. Szylińska et al. confirmed this relationship in cardiac surgery [35]. However, data from the literature indicate that, today, the Comprehensive Geriatric Assessment (CGA) should be used in the preoperative assessment of elderly patients because it more accurately identifies patients at higher risk of complications than traditional assessment. The CGA not only evaluates comorbidities but also includes assessments of frailty, sarcopenia, nutritional status, and functional status, which are crucial in predicting postoperative outcomes [36,37].

The cognitive reserve before surgery is most often determined only subjectively, rarely using available tests. A recent review of the literature showed that the preoperative MMSE score can significantly predict POD after a variety of cancer surgeries [38]. However, a specific optimal MMSE cutoff score that would indicate the possible occurrence of POD in patients of different ages has not yet been determined. In our study, the preoperative MMSE score was an independent risk factor, and the cutoff value was 25.5. Patients with a score lower than this had a significantly higher incidence of POD. Patients with mental/neurological disorders were not included in this study to avoid confounding factors. Patients with pre-existing dementia, severe cognitive impairment, or active psychiatric disorders have particularly high baseline risk for developing delirium. Therefore, we initially excluded patients with severe cognitive impairment (MMSE ≤ 17). In this study, the preoperative MMSE score was an independent risk factor for POD development. ROC curve analysis for MMSE scores among the included participants determined a cutoff value ≤ 25.5. Patients with a score lower than this had a significantly higher incidence of POD in study cohort. Wu et al. investigated the association between the preoperative MMSE score and POD in young/old (≤80 years) and old/old (>80 years) patients who underwent an orthopedic procedure. The optimal cutoff MMSE scores associated with POD for young/old and old/old patients were different (18.4 vs. 21.4) [39]. Large et al. also found that the preoperative MMSE score had a significant role in the development of POD [29]. However, two systematic literature reviews investigating risk factors for POD after urological surgery did not show the significance of the preoperative MMSE score [14,15].

This study showed the importance of two intraoperative variables, namely, blood transfusion and intraoperative hypotension. However, the amount of administered transfusion was not significant. The results of earlier research are different. One study demonstrated that intraoperative blood transfusion is an independent risk factor for POD after major noncardiac surgery, and intraoperative transfusion of more than 1000 mL was the strongest predictor of delirium [40]. This effect is most often explained by the impact of blood transfusion on the activation of systemic inflammation and cytokine dysregulation that can cause POD. However, in a study that included 3967 patients undergoing spinal fusion surgery, intraoperative and postoperative transfusion had no effect on POD incidence. In this study, 234 patients (5.9%) developed POD. Of the patients who developed POD, 119 (50.9%) received intraoperative transfusion and 42 (17.9%) received postoperative transfusion. Furthermore, 1032 (27.6%) patients without POD received intraoperative transfusion, and 431 (11.5%) received postoperative transfusion. Statistical analysis showed that neither perioperative transfusion nor transfusion volume had a statistically significant effect on the incidence of POD [41].

Multivariate analysis in our study showed that intraoperative hypotension is an independent risk factor for POD. As many as 20 out of 26 patients with POD had at least one episode of intraoperative hypotension during surgery. This is a well-established risk factor for major adverse events and might contribute to the development of POD through inadequate cerebral perfusion, especially in elderly patients with compromised cerebral autoregulation [42]. Its influence on POD occurrence mostly depends on its duration. Hypotension lasting >5 min can cause cerebral hypoperfusion, leading to the development of POD [43]. A large retrospective cohort study that included 316,717 surgical patients showed that intraoperative MAP < 55 mmHg was associated with POD, and this association was duration-dependent [44].

Postoperative pain intensity was found to be a risk factor for POD in the present study, which was similar to previous studies [45]. The best cutoff value of postoperative NRS score in this cohort was 4.85. Acute pain is common after surgery and can often be severe after open major procedures. This study showed that patients with POD more often had moderate or severe postoperative pain. Surgery tissue trauma and inflammation underlie pain experience. However, severe acute postoperative pain itself can increase inflammation and neuroinflammation, and trigger neurotransmitter release, potentially contributing to acute cognitive dysfunction. On the other hand, opioids are most commonly used in the treatment of severe postoperative pain. Their use may contribute to POD occurrence due to their effect on receptors in the central nervous system. Furthermore, the brain of an elderly patient is particularly sensitive to their effects. In a prospective cohort study that included 581 patients scheduled for major noncardiac surgery, it was determined that a high intensity of postoperative pain, as well as the use of high doses of opioids, increased the risk of POD in all patients, even in those with low preoperative risk factors [46]. A recent systematic review of the literature that included 30 studies, involving a total of 9213 adults undergoing noncardiac surgery, showed that postoperative pain may be a risk factor for developing POD, but there is low-certainty evidence that higher pain intensity may be associated with a clinically significant increase in the occurrence of POD [47]. In the European Society of Anesthesiology guideline for postoperative delirium, one of the recommendations is regular adequate postoperative pain assessment and treatment. Careful titration of anesthesia guided by monitoring and adequate perioperative pain control are the most effective evidence-based strategies to reduce the risks of POD [48].

The present study has potential limitations. This is a single-center study, and therefore, our results may not be generalizable. The study included only one gender. The reasons for this limitation are methodological and related to the surgical intervention. Most of the surgical procedures mentioned in the study (radical prostatectomy, cystectomy, nephrectomy and nephroureterectomy) are predominantly male procedures in the included age group (especially prostatectomy). By limiting the sample to one gender (male), the variability caused by gender differences is reduced. The aim was to more precisely isolate the influence of other factors by fixing gender. Several multivariate logistic regression results show very wide confidence intervals that may reflect a limited number of POD events and potential model overfitting. Therefore, our results should be taken with caution and verified in a sample with a larger number of POD events. Also, since the number of patients with delirium was relatively low, the sample size may be too small to identify all significant associations related to POD. Some relevant information was not included, such as medication, preoperative and postoperative laboratory tests, frailty, sarcopenia, nutritional status, and functional status, which could have had confounding effects on our analysis and findings.

## 5. Conclusions

The study findings highlight the multifactorial nature of POD. Older age, COPD, mild/moderate alcohol consumption, MMSE score lower than 25.5, intraoperative hypotension, and moderate to severe postoperative pain can help to identify elderly patients at increased risk of delirium. Considering that some of the mentioned risk factors can be modified, it is necessary to emphasize that the prevention of POD is possible and should be one of the treatment priorities.

## Figures and Tables

**Table 1:** Demographics and clinical characteristics.

Characteristic	Total (*n* = 162)	Without POD (*n* = 136)	With POD (*n* = 26)	*p* Value *
Age (Years)				<0.001
Mean (SD)	67.41 (7.38)	66.29 (7.21)	73.31 (5.18)	
Median (Range)	68 (50–84)	66.5 (50–82)	73.5 (64–84)	
Place of Residence				0.008
Rural	41 (25.31%)	29 (21.32%)	12 (46.15%)	
Urban	121 (74.69%)	107 (78.68%)	14 (53.85%)	
Marital Status				0.884
Single	4 (2.47%)	4 (2.94%)	0 (0%)	
Married	131 (80.86%)	108 (79.41%)	23 (88.46%)	
Divorced	13 (8.02%)	12 (8.82%)	1 (3.85%)	
Widower	14 (8.64%)	12 (8.82%)	2 (7.69%)	
Educational Level				0.256
Elementary school	14 (8.64%)	9 (6.62%)	5 (19.23%)	
High school	88 (54.32%)	76 (55.88%)	12 (46.15%)	
Junior college	30 (18.52%)	24 (17.65%)	6 (23.08%)	
College	26 (16.05%)	23 (16.91%)	3 (11.54%)	
Graduate school	4 (2.47%)	4 (2.94%)	0 (0%)	
BMI (kg/m^2^)				0.272
Mean (SD)	27.04 (3.89)	27.2 (3.88)	26.2 (3.93)	
Median (Range)	26.9 (17.7–39.3)	27.2 (19.2–39.3)	26 (17.7–34.6)	
Smoking				0.876
Yes	57 (35.19%)	47 (34.56%)	10 (38.46%)	
No	105 (64.81%)	89 (65.44%)	16 (61.54%)	
Alcohol Consumption				<0.001
Mild/Moderate	63 (38.89%)	41 (30.15%)	22 (84.62%)	
Never	99 (61.11%)	95 (69.85%)	4 (15.38%)	
Number of Comorbidities				<0.001
Mean (SD)	1.91 (1.4)	1.72 (1.29)	2.88 (1.56)	
Median (Range)	2 (0–7)	1 (0–7)	2.5 (1–7)	
Hypertension				0.137
Yes	124 (76.54%)	101 (74.26%)	23 (88.46%)	
No	38 (23.46%)	35 (25.74%)	3 (11.54%)	
Atrial Fibrillation				0.034
Yes	18 (11.11%)	12 (8.82%)	6 (23.08%)	
No	144 (88.89%)	124 (91.18%)	20 (76.92%)	
Ischemic Heart Disease				0.690
Yes	11 (6.79%)	9 (6.62%)	2 (7.69%)	
No	151 (93.21%)	127 (93.38%)	24 (92.31%)	
Diabetes Mellitus				0.642
Yes	32 (19.75%)	26 (19.12%)	6 (23.08%)	
No	130 (80.25%)	110 (80.88%)	20 (76.92%)	
COPD				0.001
Yes	22 (13.58%)	13 (9.56%)	9 (34.62%)	
No	140 (86.42%)	123 (90.44%)	17 (65.38%)	
Chronic Kidney Disease				0.071
Yes	45 (27.78%)	34 (25%)	11 (42.31%)	
No	117 (72.22%)	102 (75%)	15 (57.69%)	

* Wilcoxon rank-sum test, Pearson chi-squared test, Fisher exact test. POD—Postoperative delirium; SD—Standard deviation; BMI—Body mass index; COPD—Chronic obstructive pulmonary disease.

**Table 2:** Perioperative characteristics.

Characteristic	Total (*n* = 162)	Without POD (*n* = 136)	With POD (*n* = 26)	*p* Value *
Preoperative MMSE				<0.001
Mean (SD)	27.9 (2.17)	28.27 (1.9)	25.96 (2.51)	
Median (Range)	28 (20–30)	29 (21–30)	25 (20–30)	
Intraoperative EEG monitoring				0.271
Yes	72 (44.44%)	63 (46.32%)	9 (34.62%)	
No	90 (55.56%)	73 (53.68%)	17 (65.38%)	
Type of surgery				0.020
Radical cystectomy	45 (27.78%)	33 (24.26%)	12 (46.15%)	
Radical nephrectomy	51 (31.48%)	41 (30.15%)	10 (38.46%)	
Nephroureterectomy	11 (6.79%)	10 (7.35%)	1 (3.85%)	
Radical prostatectomy	55 (33.95%)	52 (38.24%)	3 (11.54%)	
Surgery duration (min)				0.223
Mean (SD)	172.2 (65.69)	168.6 (62.03)	191 (81.11)	
Median (Range)	170 (60–480)	167.5 (60–420)	175 (100–480)	
Intraoperative hypotension				<0.001
Yes	68 (41.98%)	48 (35.29%)	20 (76.92%)	
No	94 (58.02%)	88 (64.71%)	6 (23.08%)	
Estimated blood loss (mL)				0.066
Mean (SD)	395.6 (303.87)	374.8 (293.21)	506 (340.75)	
Median (Range)	300 (50–1400)	300 (50–1400)	500 (50–1200)	
Intraoperative blood transfusion				0.018
Yes	39 (24.07%)	28 (20.59%)	11 (42.31%)	
No	123 (75.93%)	108 (79.41%)	15 (57.69%)	
Intraoperative blood transfusion amount (mL)				0.754
Mean (SD)	343.5 (119.26)	332.3 (110.17)	371.8 (141.59)	
Median (Range)	285 (235–610)	285 (235–575)	285 (245–610)	
Postoperative pain intensity (NRS)				<0.001
Mean (SD)	4.19 (1.74)	3.89 (1.69)	5.73 (1.01)	
Median (Range)	4.3 (0–8.7)	3.7 (0–8.7)	5.7 (3.7–7.3)	

* Wilcoxon rank-sum test, Pearson chi-squared test, Fisher exact test. POD—Postoperative delirium; MMSE—Mini mental state examination; SD—Standard deviation; EEG—Electroencephalography; NRS—Numerical rating scale.

**Table 3:** ROC cutoff values and postoperative delirium.

Demographics and Clinical Data	Total (*n* = 162)	Without POD (*n* = 136)	With POD (*n* = 26)	*p* Value *
Age (years)				<0.001
<67.5 years	80 (49.38%)	77 (56.62%)	3 (11.54%)	
≥67.5 years	82 (50.62%)	59 (43.38%)	23 (88.46%)	
Number of comorbidities				0.001
<1.5	74 (45.68%)	70 (51.47%)	4 (15.38%)	
≥1.5	88 (54.32%)	66 (48.53%)	22 (84.62%)	
Preoperative MMSE				<0.001
≤25.5	26 (16.05%)	12 (8.82%)	14 (53.85%)	
>25.5	136 (83.95%)	124 (91.18%)	12 (46.15%)	
Postoperative pain intensity (NRS)				<0.001
<4.85	104 (64.2%)	99 (72.79%)	5 (19.23%)	
≥4.85	58 (35.8%)	37 (27.21%)	21 (80.77%)	

* Fisher exact test, Pearson chi-squared test. POD—Postoperative delirium; MMSE—Mini mental state examination; NRS—Numerical rating scale.

**Table 4:** Univariate and multivariate logistic regression analysis of independent risk factors for postoperative delirium.

Characteristics	Logistic Regression for Pts with POD			
	Univariate		Multivariate	
	OR (95% CI)	Wald Test	OR (95%CI)	Wald Test
Age				
≥67.5:<67.5	10.0 (2.87–34.92)	3.05 × 10^−4^	6.30 (1.12–35.33)	0.036
Place of residence				
Rural/Urban	3.2 (1.32–7.57)	9.77 × 10^−3^	-	0.37 ^#^
Number of comorbidities				
2:0 and 1	5.8 (1.91–17.83)	1.97 × 10^−3^	-	0.14 ^#^
COPD				
Yes/No	5.0 (1.86–13.48)	1.42 × 10^−3^	14.52 (2.09–100.94)	0.007
Atrial fibrillation				
Yes/No	3.1 (1.04–9.20)	0.04	-	0.52 ^#^
Alcohol consumption				
Mild or Moderate/Never	3.35 (1.39–8.09)	9.48 × 10^−6^	20.73 (3.67–117.03)	0.001
MMSE score				
≤25.5:>25.5	12.1 (4.56–31.88)	5.23 × 10^−7^	10.14 (1.83–56.05)	0.008
Intraoperative hypotension				
Yes/No	6.1 (2.30–16.25)	2.85 × 10^−4^	15.18 (2.68–85.80)	0.002
Intraoperative blood transfusion				
Yes/No	2.8 (1.17–6.83)	0.02	-	0.24 ^#^
NRS pain score				
≥4.85:<4.85	11.2 (3.95–31.98)	5.79 × 10^−6^	17.58 (3.04–101.49)	0.001

^#^ Likelihood ratio test. POD—Postoperative delirium; OR—Odds ratio; CI—Confidence interval; COPD—Chronic obstructive pulmonary disease; MMSE—Mini mental state examination; NRS—Numerical rating scale.

## Data Availability

The raw data supporting the conclusions of this article will be made available by the authors on request.

## Supplementary Materials

The following supporting information can be downloaded at: https://www.mdpi.com/article/10.3390/diagnostics15243165/s1, Table S1: Results of the ROC analysis for predicting postoperative delirium; Figure S1: ROC curves and the best cutoff values for predicting postoperative delirium: (A) age in years; (B) number of comorbidities; (C) preoperative MMSE (Mini mental state examination) score; (D) postoperative NRS (Numerical rating scale) score.
