# Supplementary material for: Incidence and Perioperative Risk Factors for Postoperative Delirium After Major Urological Surgery
Source: Diagnostics (Basel). 2025 Dec 11;15(24):3165. doi: 10.3390/diagnostics15243165 (PMC12731831; doi:10.3390/diagnostics15243165)
Supplement: Supplementary file 1 [file diagnostics-15-03165-s001.zip › Supplement2.pdf]

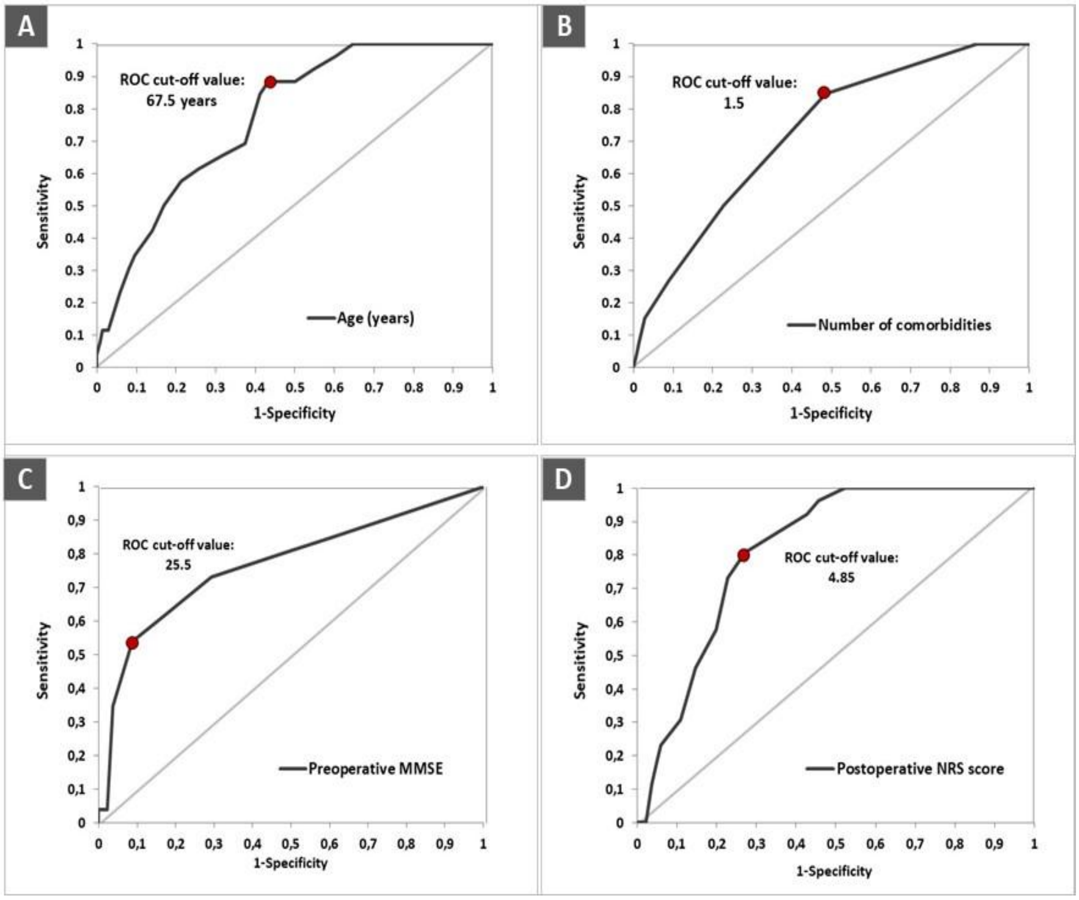

Figure S1. ROC curves and the best cutoff values for predicting postoperative delirium: (A) age in years; (B) number of comorbidities; (C) preoperative MMSE (Mini mental state examination) score; (D) postoperative NRS (Numerical rating scale) score.
