# Supplementary material for: Incidence and Perioperative Risk Factors for Postoperative Delirium After Major Urological Surgery
Source: Diagnostics (Basel). 2025 Dec 11;15(24):3165. doi: 10.3390/diagnostics15243165 (PMC12731831; doi:10.3390/diagnostics15243165)
Supplement: Supplementary file 1 [file diagnostics-15-03165-s001.zip › Supplement1.pdf]

Table S1. Results of the ROC analysis for predicting postoperative delirium.

Characteristic	Results of the ROC analysis				
	AUC ROC[a] (95%CI)	p value[b]	Best cutoff[c]	Sensitivity (95%CI)	Specificity (95%CI)
**Age (years)**	77.3% (68.8-85.9%)	$1.8*10^{-6}$	67.5	88.5% (73.1-100%)	56.6% (48.5-64.7%)
**No. of comorbidities**	72.7% (63.2-82.2%)	$2.2*10^{-4}$	1.5	84.6% (69.2-96.2%)	51.5% (42.6-60.3%)
**Preoperative MMSE**	76.8% (65.4-88.1%)	$1.9*10^{-6}$	25.5	53.8% (34.6-73.1%)	91.2% (86.0-95.6%)
**Postoperative NRS**	81.7% (74.8-88.5%)	$2.6*10^{-7}$	4.85	80.8% (65.4-96.2%)	72.8% (65.4-80.2%)

[a]Area Under the ROC Curve (DeLong method); [b]Likelihood ratio test for AUC ROC; [c]Value with maximum sensitivity and specificity. CI – Confidence interval; MMSE – Mini mental state examination; NRS – Numerical rating scale.
